# Magnetic domain orientation in magnetite Fe_3_O_4_ nanoparticles coated with natural extracts from *Syzygium aromaticum*, *Illicium verum* or *Lippia graveolens*

**DOI:** 10.1039/d5ra08815a

**Published:** 2026-03-30

**Authors:** Nidia Esther Moreno-Cabrera, Mauricio D. Carbajal-Tinoco, J. Santoyo-Salazar

**Affiliations:** a Departamento de Investigación y Estudios Multidisciplinarios, Programa de Doctorado en Nanociencias y Nanotecnología, Centro de Investigación y de Estudios Avanzados del Instituto Politécnico Nacional, Cinvestav Mexico City 07360 Mexico nidia.moreno@cinvestav.mx; b Departamento de Física, Centro de Investigación y de Estudios Avanzados del Instituto Politécnico Nacional, Cinvestav Mexico City 07360 Mexico jaime.santoyo@cinvestav.mx

## Abstract

This work contributes with the development of an alternative direct processing of magnetite nanoparticles (<20 nm) coated with polyphenols (Fe_3_O_4_NPs@polyphenols), which could be regarded as starting point from Fe–OH, and Fe–CH_3_O to bind organic groups for the formulation or attaching of multifunctional systems. Fe_3_O_4_NPs@polyphenols have shown reproducible uniaxial Single Magnetic Domains (SMDs) and superparamagnetic performance, ideal directional core as single magnetic domains with agglomeration in low dimension and promising development as nano-stirring, nano-carriers or nano-trigger formulations. The synthesis and coating were performed using an improved Massat's co-precipitation route and adding polyphenol extracts from (1) *Syzygium aromaticum*, (2) *Illicium verum*, or (3) *Lippia graveolens*. The phenol chains allowed the synthesis of SPIONs with particle sizes ranging from 3.09 to 7.31 nm, coated with a polyphenol thin layer of thickness *t* ≈ 1–3 nm. pH values of 13–14 and temperatures within 70–80 °C were necessary to obtain Fe_3_O_4_NPs@polyphenols. Fe_3_O_4_NPs@polyphenols exhibited an inverse spinel structure and small lattice distortion related to γ-Fe_2_O_3_ formation over their surface as well as superparamagnetic response, phenol bonding Fe–OH on their interphase, and directional behavior with effective uniaxial magnetic response as SMDs compass.

## Abstract 2

Direct polyphenol attachment on the surfaces of Fe_3_O_4_ nanoparticles <10 nm was done with extracts of *S. aromaticum*, *I. verum*, or *L. graveolens*. These exhibited effective superparamagnetic uniaxial magnetic response, plus phenol bonding Fe–OH action.

## Graphical abstract

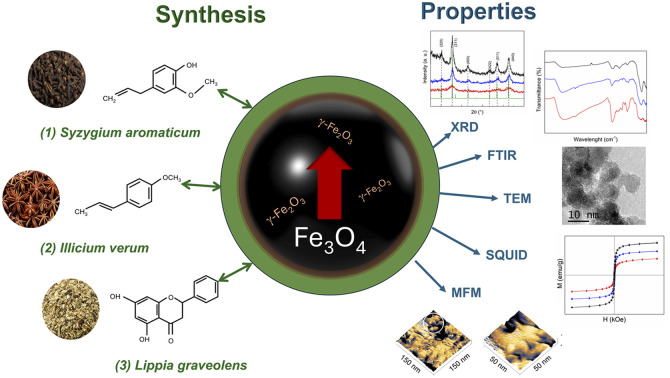

## Introduction

Magnetite (Fe_3_O_4_) nanoparticles (MNPs) belong to the class of superparamagnetic iron oxide nanoparticles (SPIONs) when their particle size is less than 20 nm. They have been investigated as promising nanocarriers, nanostirrers, nanotriggers, and heating nanogenerators for biomedical applications, such as drug release, diagnosis, and therapy. MNPs exhibit a ferromagnetic moment owing to the electronic interaction between Fe^3+^ and Fe^2+^ ions at the octahedral positions in their inverse spinel structures. Fe^3+^ and Fe^2+^ contribute with double exchange tuning the directional single magnetic domains (SMDs) properties in agreement with the superparamagnetic size (<20 nm). Many studies on MNPs have shown their high saturation magnetization (*M*_S_) and low coercivity (*H*_C_). However, *M*_S_ can be reduced by an organic coating depending on the size and thickness of the coating layer, for instance, Alvarado-Noguez *et al.* reported a reduction in *M*_S_ from 76.0 emu g^−1^ to 8.81 emu g^−1^ (ref. 1) for semi-spherical nanoparticles in the range of 7–16 nm coated with a 3 nm thick turmeric extract layer. In addition, Ramirez *et al.* obtained iron oxide nanoparticles, coated with *Vanilla planifolia* with an amorphous surface layer thickness *t* ≈ 5–9 nm and an average particle size of 12 nm, and reported a reduction in *M*_S_ from 68.16 emu g^−1^ to 59.45 emu g^−1^.^2^ The organic coating acts as an antiferromagnetic barrier, causing disorder on the surface of the superparamagnetic nanoparticles.^3^ Magnetometry techniques, such as SQUID and VSM, could reveal the essential magnetization properties of MNPs, but local magnetic interactions at the nanometric scale remain underexplored, particularly for the green synthesis routes of nanoparticles, where surface modifications and aggregation might affect magnetic interactions. In this regard, magnetic force microscopy (MFM) measurements offer an efficient approach to investigate local magnetic responses with nanometric resolution, enabling the analysis of the magnetic interactions of single MNPs or small aggregates. MNPs have been coated with polyphenols to act in biological media, providing biocompatibility, biodegradability, low toxicity, thermal, chemical, and colloidal stability, and surface modification capability.^4–8^ These features make them promising candidates for therapeutic and diagnostic applications, such as in magnetic hyperthermia, targeted drug and gene delivery, magnetic bio-separation, and tissue engineering; they also find applications as biosensors, anti-bacterials, and as contrast agents in magnetic imaging.^9–11^ Thus, some formulations with SPIONs have been approved by the US Food and Drug Administration (FDA).^12–15^ Currently, Feraheme® is the only SPION product approved by the FDA since 2009 for the treatment of iron deficiency anaemia in adult patients with chronic kidney disease in the US (https://www.accessdata.fda.gov). However, ferumoxytol, the active ingredient of Feraheme®, is also under active clinical trials in the US (https://www.clinicaltrials.gov) as an imaging agent for the detection of primary and metastatic hepatic cancer (clinical trial ID: NCT04682847), bladder cancer (clinical trial ID: NCT04369560), and lymphoma and sarcoma cancer in children (clinical trial ID: NCT01542879). In addition, a clinical trial evaluates pharmacologic ascorbate and ferumoxytol combined with concomitant temozolomide and radiation therapy for the treatment of glioblastoma multiforme in adults (clinical trial ID: NCT04900792).

Although recent decades have seen research on MNPs highlighting their use in biomedical applications, mainly in cancer-based therapies, the surfaces of iron oxide nanoparticles have been shown to generate reactive oxygen species (ROS).^16^ Hence, high concentrations of MNPs can induce oxidative stress in molecules such as proteins, lipids, and DNA, triggering structural modifications of proteins, nucleic acid mutations, lipid peroxidation, and DNA strand breakage.^17^ In addition, bare magnetite nanoparticles have poor colloidal stability because the attractive forces between the nanoparticles become dominant over the repulsive forces, resulting in agglomerate formation. The key to achieving a stabilized system is to balance the interactions between the nanoparticles by favouring repulsive forces. Superficial functionalization, which involves the formation of a physical barrier that creates steric repulsion, is an approach to achieve colloidal stabilization. Steric forces prevent nanoparticles from getting too close to each other, ensuring that the dispersion is stable.^17^ In addition to preventing the agglomeration of SPIONs, surface modification improves the surface catalytic activity and chemical and mechanical properties and increases solubility and biocompatibility.^10,11^

Green biosynthesis and wet chemistry methods, such as co-precipitation, have gained relevance in recent years for the functionalization of magnetic nanoparticles with organic molecules. Among the wide range of organic materials, plant-based synthesis is preferred because of the presence of polyphenol compounds that can reduce metal ions and act as stabilizing agents during the synthesis of nanoparticles.^18,19^ Polyphenols are compounds with one or more hydroxyl (–OH) groups attached to an aromatic ring structure. Based on their origin, biological function, and chemical structure, polyphenols can be classified into phenolic acids, coumarins, flavonoids, stilbenes, tannins, and lignans.^20^ The main biological function of polyphenols is their antioxidant activity, as they can neutralize free radicals by donating an electron or hydrogen atom, suppressing the generation of free radicals and thus reducing the rate of oxidation by inhibiting the formation of reactive oxygen species. Therefore, polyphenols present in fruits, vegetables, seeds, seasonings, and nuts have been linked to the prevention and treatment of chronic diseases, displaying anti-inflammatory, anti-cancer, antibacterial, and immune system-promoting effects, cardioprotective and neuroprotective effects, and skin protection from UV radiation.^21^

Direct polyphenol attachment to the surfaces of MNPs efficiently integrates the benefits of plant extracts with the magnetic properties of the nanoparticles. In this regard, the covalent bond established between the hydroxyl groups and iron ions (Fe–OH) on the surface of the nanoparticles promotes the colloidal stability of the nanoparticles through their bioactivity, which simultaneously acts with their medicinal and antioxidant properties, enhancing the biocompatibility and minimizing the cytotoxicity of MNPs. In this study, the following polyphenols were attached to the MNP surface because of their high polyphenolic content: (1) clove (*Syzygium aromaticum*), a tree member of the Myrtaceae family, and its aromatic flower buds represent one of the major sources of phenolic compounds. The plant material (100 g) contained 15 188 mg of total polyphenol content, as determined by high-performance liquid chromatography.^22^ The aqueous extract of clove had a total content of polyphenols and flavonoids of 239.58 ± 0.70 mg gallic acid equivalents and 65.67 ± 0.01 mg catechin equivalents per gram of dried extract.^23^ According to the Phenol-Explorer database, the most prevalent polyphenols in clove buds are flavonoids, phenolic acids, and hydroxyphenylpropenes, including quercetin, gallic acid, acetyl eugenol, and eugenol.^24^ These phytochemicals have been extensively studied owing to their antimicrobial, antibacterial, anti-inflammatory, antioxidant, antifungal, antiviral, antiparasitic, antiseptic, and anticarcinogenic properties.^25–30^ Eugenol is the main bioactive compound of clove, with a concentration between 9381.70 and 14 650.00 mg per 100 g,^24^ and has a wide range of therapeutic potential against several diseases, such as hypercholesterolemia, hyperglycemia, depression, Alzheimer's disease, and cancer.^31^ Several experimental studies have shown that eugenol possesses anticancer properties against different types of cancer, such as human promyelocytic leukaemia cells (HL-60), lung cancer adenocarcinoma cells (A549), colorectal cancer (CRC) cell lines (HCT116 and HT-29), primary melanoma cell line (G361), MCF-7 human breast cancer cells, PC3 adenocarcinoma, A-375 and G361 human melanoma cells, human cervical cancer (HeLa) cells, and the inhibition of the TGF-β/SMAD4 pathway in gastric cancer metastasis.^32,33^ Quercetin has demonstrated promising therapeutic effects against several illnesses, including diabetes, hypertension, cardiovascular disease, Alzheimer's disease, Parkinson's disease, tuberculosis and cancer.^34^*In vitro* and *in vivo* studies have shown the antitumor activity of pure quercetin against diverse types of cancer. These experimental investigations reported the inhibition of cell viability and the induction of cell apoptosis in gastric cancer cell lines AGS and MKN45, hepatic cancer HepG2 cell lines, lung cancer cell lines (A549 and H69), human oesophageal cancer cell line Eca109, human colon cancer cell lines (Colo-320 and Colo-741), and human oral cancer cell lines (HSC-6 and SCC9).^35^ Among the phenolic acids present in clove, gallic acid is found at the highest concentration (17.47–783.50 mg/100 g fresh weight).^24^ The anti-cancer activity of gallic acid has been tested in different study models, including glioblastoma cells in rats, DU145 prostate cancer cells in mice, A375S2 human melanoma cells in mice, breast cancer MCF-7 human cell line, non-small-cell lung cancer (NSCLC) in human cells and rats, colon cancer in rats, human bladder cancer T24 cell line, and leukaemia and its resistant sublines (HL60 cell HL60/VINC HL60/M2) in human cells.^36^ (2) *Illicium verum*, better known as star anise, is a culinary spice with medicinal uses in the treatment of cramping pain, flatulence, spasms, and rheumatism.^37^ Quantitative analysis by high-performance liquid chromatography (HPLC) demonstrated that 100 g of star anise contains 5460 mg of total polyphenol content.^24^ Additionally, the polyphenols in *I. verum* extracts exhibit various biological activities, including antibacterial, antifungal, antioxidant, anti-inflammatory, insecticidal, and antidiarrheal activities.^37,38^ The main phytochemicals present in star anise are hydroxyphenylpropenes (*trans*-anethole) and phenolic acids (protocatechuic and caffeic acids), with the most prevalent being *trans*-anethole, with an average content of 5407.90 mg/100 g fresh weight (FW).^24^ Among the reported therapeutic effects of *trans*-anetholes are antibacterial, antifungal, anti-inflammatory, anti-obesity, and anticarcinogenic activities.^39^ In a study carried out by Harakeh *et al.*, the inhibitory effect of the *trans*-anethole on the proliferation of HepG2, HeLa, and MCF-7 cells was evaluated, and it was found to exhibit potent growth-inhibitory activity. HepG2 cells also showed increased apoptotic cell death.^40^ Caffeic acid has been reported to strongly modulate apoptosis and autophagy in cancer cells, thereby altering their proliferative capacity and survival, particularly in MCF-7, multiple myeloma, leukaemia (K562), and osteosarcoma (MG-63) cells.^41^ (3) Mexican oregano (*Lippia graveolens*) is an aromatic spice widely produced and used in Mexican gastronomy, in addition to its medicinal use in the treatment of diseases associated with inflammatory responses of the respiratory and digestive systems, headaches, and rheumatism.^42^*L. graveolens* is rich in polyphenols, with a total content of 2919 mg per 100 g of dried plant.^22^ This spice includes 17 different phytochemicals divided into five types of flavonoids (dihydrochalcones, dihydroflavonols, flavanones, flavones, and flavonols).^24^ The major polyphenolic compounds are pinocembrin, naringenin, and luteolin 7-*O*-glucoside with average concentrations of 499.33, 372.00, and 297.67 mg/100 g fresh weight, respectively.^24^ These chemical compounds have been shown to exert pharmacological effects, including antioxidant, antibacterial, anti-inflammatory, antifungal, antidiabetic, antithrombotic, neuroprotective, and cardioprotective effects.^43–46^ Regarding anticancer and antitumor properties, several studies have been conducted to date. The inhibitory effects of pinocembrin on the proliferation of two HCC cell lines, HepG2 and Li-7, were observed.^47^ In another study, this flavonoid decreased the viability of A549 lung cancer cells at concentrations of 100, 150, and 200 µg, and the apoptosis of cells was enhanced upon pinocembrin exposure.^48^ It has also been reported to have inhibitory and apoptosis-inducing effects against a wide variety of cancer types, including breast, liver, lung, prostate, pancreatic, cervical, brain, oral, skin, colorectal, bladder, and bone cancer.^49^ An *in vitro* study by Ho *et al.* revealed that luteolin-7-*O*-glucoside (LUT7G) significantly reduced the proliferation of NPC cell lines (NPC-039 and NPC-BM of nasopharyngeal carcinoma) and activated apoptotic processes.^50^ In a further investigation, the anticarcinogenic potential of the phytocompound luteolin7-*O*-glucoside, isolated from the leaves of *Ophiorrhiza mungos* Linn, was studied; the results revealed that LUT7G induced apoptosis by scavenging ROS and suppressing the expression of β-catenin in COLO 320 DM cells.^51^

In this study, we report the viability of direct MNP coating with polyphenols derived from aqueous extracts of (1) *Syzygium aromaticum*, (2) *Illicium verum* or (3) *Lippia graveolens*. We present a viable synthesis route for Fe_3_O_4_NPs@polyphenols, and their physical properties characterization, such as superparamagnetic behavior, favour the directional nanoparticle interaction under the application of an external magnetic field. As a result, SQUID measurements combined with MFM measurements reveal a comprehensive examination of the global magnetic properties and local magnetic response of Fe_3_O_4_NPs@polyphenols. In addition, the pharmacological properties of the phenolic groups attached to iron ions on the nanoparticle surface can be exploited because of their antitumor properties and their ability to provide linkers for the development of multifunctional platforms that combine both magnetic responsiveness and therapeutic potential.

## Experimental section

### Chemicals and raw materials

Ferric dichloride tetrahydrate (FeCl_2_·4H_2_O) (reagent grade, p.a., and ≥99%) was purchased from J. T. Baker, Inc., and ferric trichloride hexahydrate (FeCl_3_·6H_2_O) (reagent grade, p.a., and ≥97%) was obtained from Sigma Chemical Co. Potassium hydroxide (KOH) was obtained from Merck, and hydrochloric acid (HCL) was obtained from Sigma-Aldrich. Natural clove buds, star anise pods, and Mexican oregano leaves were purchased from a local market in Mexico City, Mexico. Dried spices were used because they have been reported to improve the extraction process and enhance the antioxidant properties of extracts compared with non-dried leaves.^52^

### Preparation of aqueous extract

Three natural extracts were prepared, one for each spice, and were processed as described below. The spice (1.0 g) was mixed with 200 mL of distilled water. The mixture was heated at 80 °C and stirred magnetically at 300 rpm for 1 h and 30 min. The extract was then cooled and stored for future use.

### Synthesis of Fe_3_O_4_NPs@polyphenols

Superparamagnetic iron oxide nanoparticles (SPIONs) were synthesized using the chemical co-precipitation technique of iron salts^53^ with the incorporation of direct polyphenol coatings on the surfaces of the magnetic nanoparticles. Due to their colloidal nature, it is important to control the temperature (70–80 °C), pH (13–14) and volume of polyphenol extracts to obtain SPIONs of <20 nm. Magnetite nanoparticle formation was carried out using a stoichiometric mixture of 2 : 1 (Fe^3+^/Fe^2+^) of ferric hydroxide and ferrous hydroxide salts in an aqueous solution. According to the chemical reaction Fe^2+^ + 2Fe^3+^ + 8OH^−^ → Fe_3_O_4_ + 4H_2_O, the formation of nanoparticles was expected at pH values of 10–14.^54^

A starting solution was prepared with 54 mL of distilled water and 10 mL of hydrochloric acid at a purity of 37%; afterward, the solution was deoxygenated using argon gas. Subsequently, 6.96 g of FeCl_3_·6H_2_O was dissolved in 25 mL of the acidic solution, and another 6.25 mL of the solution was added to 2.52 g of FeCl_2_·4H_2_O. Both solutions were then placed under magnetic stirring at 500 rpm for 30 min. 2.5 mL of the FeCl_2_·4H_2_O solution and 10 mL of the FeCl_3_·6H_2_O solution were mixed in a stoichiometric ratio of 2Fe^3+^ : Fe^2+^ in a three-necked flask at argon atmosphere and heated until a stable temperature of 70 °C. The mixture was stirred mechanically at 150 rpm and 70 °C for 15 min. Subsequently, a 0.7 M KOH solution was added dropwise to the three-necked flask to increase the pH of the solution to an alkaline scale, allowing for the formation of black magnetic nanoparticles. Subsequently, an aqueous extract was added to the solution until the mixture reached a pH of 14. Then, 51 mL of clove, 100 mL of oregano, and 120 mL of star anise extracts were added to each sample. The final solutions were stirred for 15 min and cooled to room temperature. The three syntheses were precipitated by magnetic decantation and washed three times with a mixture of distilled water and ethanol to eliminate organic residuals. The dispersed Fe_3_O_4_NPs@polyphenols were washed twice with distilled water and lyophilized to obtain powdered nanoparticles, which were stored under vacuum to avoid oxidation.

### Characterization

XRD characterization of the nanoparticles was performed using a RIGAKU Smart Lab X-ray diffractometer (Rigaku Corporation, Japan) supplied with a Cu Kα source of 1.5424 Å wavelength operating at an acceleration voltage of 35 kV and a current of 25 mA with a step rate of 0.02° in the 2*θ* range of 20°–75°. The experimental diffraction patterns were analyzed using the PowderCell 2.3 software to estimate the average crystallite size of the powder samples. The diffractograms were compared with the database powder diffraction file, JCPDS-PDF PDF-19-0629. The bands of the functional groups of the inorganic and organic substances of the coated nanoparticles were analyzed using an FT-IR spectrophotometer, NICOLET 6700, (Thermo Scientific, Waltham, MA, USA). The measurements were carried out in the range of 4000–450 cm^−1^ in the transmission mode. The shape and particle size of the magnetic nanoparticles were observed using a JEOL-JEM 2010 (JEOL, Tokyo, Japan) transmission electron microscope (TEM) equipped with a LaB_6_ filament at 200 kV. Each of the three samples was diluted in an ethanol solution and added to a lacy, carbon-coated, 400-mesh copper grid by dropping it. Digital Micrograph 3.57 software was used to process the TEM micrographs and determine the particle size distribution. Magnetization hysteresis loops were measured for all samples using a superconducting quantum interference device (SQUID) magnetometer model MPMS-3 (Quantum Design, USA), and the magnetic response *M*_S_ (emu g^−1^) *versus H* (Oe) was acquired with an applied magnetic field of 3 T under ambient temperature conditions. Magnetic force microscopy (MFM) images were obtained using a scanning probe microscope (SPM), JEOL-JSPM-5200 (JEOL, Tokyo, Japan). Topography analysis was performed in the tapping mode of atomic force microscopy (AFM), and the magnetic domain interaction was sensed in the MFM mode. The powder samples were flattened between two porta objects separately and pasted onto a carbon adhesive tape. Previously, the tip NSC18, Co–Cr/Al MicroMasch, with a radius of 8 nm, a coated radius of <40 nm, and a full tip cone angle of 40°, was magnetized with a neodymium magnet and used to scan over single magnetic domain (SMD) surfaces. Topography and MFM images were obtained using a distance tip-sample at *Z* and (*Z* + Δ*Z*) lift height in the range of 5–86 nm, respectively, with output signals between 0.011 and 0.025 A/V, and an applied magnetic field of *H* ≈ 5 kOe. 3D MFM mapping images and profiles were processed with Gwyddion software, version 2.58, SPM analysis.

## Results and discussion

**Fig. 1:**
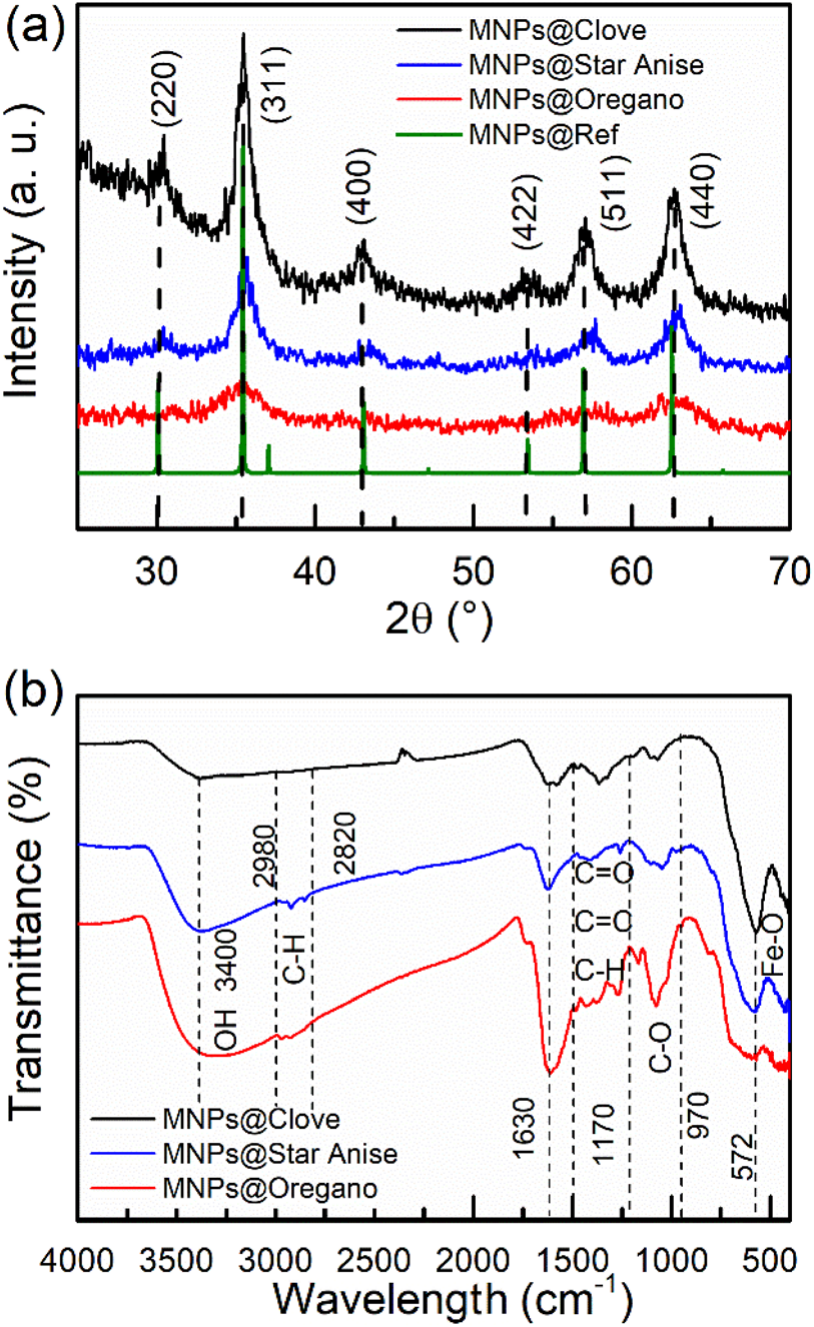
(a) XRD patterns of Fe_3_O_4_NPs@polyphenols synthesized using the extracts of *S. aromaticum* (MNPs@Clove, black line), *I. verum* (MPNs@Star Anise, blue line) and *L. graveolens* (MNPs@Oregano, red lines). (b) FTIR spectra of the three samples.

**Fig. 2:**
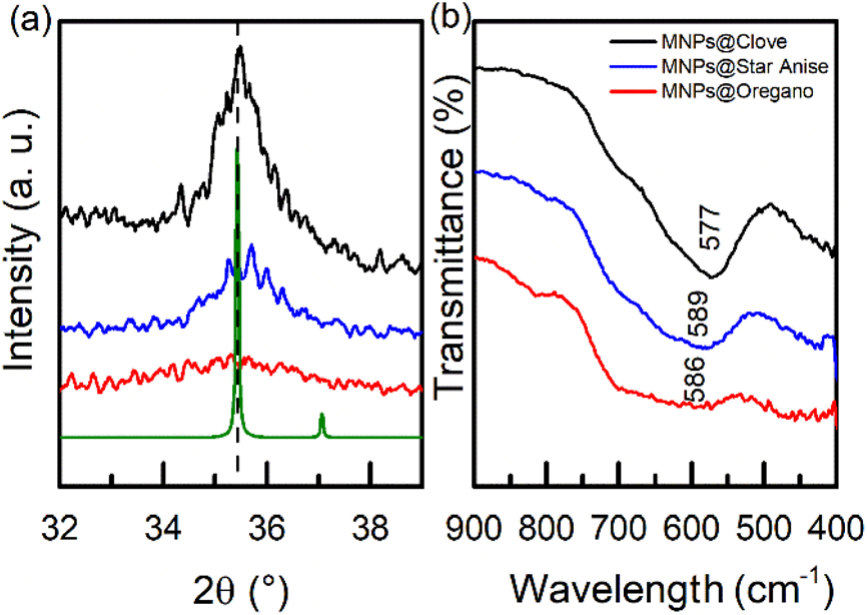
(a) Magnified view of the (311) plane diffraction from the XRD patterns of MNPs@Clove, MPNs@Star Anise, and MNPs@Oregano. (b) Magnified view of the Fe–O stretching band from the FTIR spectra of the three samples.

The three samples were labeled as MNPs@Clove (black line), MNPs@Star Anise (blue line), and MNPs@Oregano (red line), corresponding to nanoparticles with extracts of clove (*S. aromaticum*), star anise (*I. verum*), and Mexican oregano (*L. graveolens*), respectively. XRD analysis (Fig. 1a) showed the main diffraction peaks corresponding to the structure of magnetite (Fe_3_O_4_) at 2*θ* positions of 30° (220), 35.19° (311), 43° (400), 53° (422), 57° (511), and 62° (440). The (311) diffracted plane was the principal crystalline growth orientation. The main diffraction planes were related to a cubic inverse spinel structure (FCC), space group *Fd*3̄*m* (227), and lattice parameter of 8.396 Å (JCPDS card no. 19-0629, https://www.icdd.com). This structure exhibits ferromagnetic moment behavior owing to a double-exchange mechanism between divalent Fe^2+^ and trivalent Fe^3+^ ions occupying octahedral sites, and Fe^3+^ ions at tetrahedral sites exert an antiferromagnetic response.^55^ An amorphous contribution of the biomolecules covalently bonded to the iron ions on the surface of the nanoparticles was observed as a nonlinear slope in the baseline of the diffractograms; however, they did not lose their crystalline structure. Furthermore, the wide peaks are related to nanometric particle sizes. The mean crystallite sizes <*D*> were estimated in PowderCell 2.3 by matching the theoretical structure with the experimental diffraction results: <*D*> = 7.31 nm for MNPs@Clove, <*D*> = 5.30 nm for MNPs@Star Anise, and <*D*> = 3.09 nm for MNPs@Oregano. Additionally, crystallite sizes and their corresponding errors were calculated using the Debye–Scherrer equation (*K* = 0.95) from the FWHM values of the main diffraction peaks. The FWHM values were obtained from the pseudo-Voigt fitting of the diffraction peaks using the Origin Pro 2016 software. The fitting was performed only for the (311), (511) and (440) planes, as they are the most well-defined and intense peaks. For MNPs@Clove, the FWHM values were 0.953 (311), 1.269 (511) and 1.618 (440); for MNPs@Star Anise, the FWHM values were 1.570 (311), 2.676 (511) and 1.670 (440); and for MNPs@Oregano, the FWHM values were 3.258 (311), 5.697 (511), and 3.026 (440). The average crystallite size for each sample was defined as <*D*> = 7.4 ± 0.3 nm for MNPs@Clove, <*D*> = 5.32 ± 0.19 nm for MNPs@Star Anise and <*D*> = 3.11 ± 0.17 nm in the case of MNPs@Oregano. Ganapathe *et al.* investigated the effect of molarity on the structural, magnetic, and heat dissipation properties of magnetite nanoparticles. The authors have reported crystallite sizes of 3.599–7.805 nm with FWHM values in the range of 0.9694–2.103,^56^ which are consistent with the obtained values. It is important to note that the FWHM of MNPs@Oregano showed higher values because the peaks are not well-defined due to the ultra-fine crystallite size of nanoparticles. The organic layer also contributes to the scattering of the peaks, incorporating further peak broadening.^57^ The lattice parameters obtained using PowderCell 2.3 showed small distortions with respect to the magnetite lattice parameter: *a* = 8.382 Å in MNPs@Clove, *a* = 8.388 Å in MNPs@Star Anise, and *a* = 8.380 Å in MNPs@Oregano. Furthermore, the lattice parameters were determined using the Bragg equation and the (311), (511), and (440) diffraction planes. The values obtained were *a* = 8.389 ± 0.0009 for MNPs@Clove, *a* = 8.364 ± 0.0017 for MNPs@Star Anise, and *a* = 8.386 ± 0.0066 for MNPs@Oregano. The uncertainties were calculated by propagating the standard errors of the Bragg angles derived from pseudo-Voigt peak fitting. The changes in *a* were associated with Fe^2+^ vacancies in the structure of all Fe_3_O_4_NPs@polyphenols because of surface oxidation during their processing and environmental interaction; this oxidation is evident as a slight displacement in the peaks (Fig. 2a). Consequently, a core–shell Fe_3_O_4_-γ Fe_3_O_4_ structure was observed.^2^

FTIR analysis (Fig. 1b) of the three different samples presented an iron–oxygen bond signal, characteristic of the formation of the magnetite phase, at approximately 572 cm^−1^.^58^ The fitted peak positions of the Fe–O stretching band with their associated errors were 577.12 ± 0.8138 cm^−1^ for MNPs@Clove, 589.52 ± 0.710 cm^−1^ for MNPs@Star Anise, and 586.21 ± 6.0282 cm^−1^ for MNPs@Oregano; their FWHMs were 92.59 ± 3.33 cm^−1^, 84.51 ± 1.822 cm^−1^, and 119.94 ± 32.98 cm^−1^, respectively. The FWHM of MNPs@Oregano had a larger standard error; this behavior is attributable to the intrinsic broad character of the Fe–O absorption band in magnetite nanoparticles. Small shoulders in the 600–780 cm^−1^ range appear (Fig. 2b). This suggests the presence of an oxidized γ-Fe_2_O_3_ layer at the Fe_3_O_4_NPs@polyphenol surface; it has been shown that with small-sized nanoparticles, the proportion of maghemite increases.^59^ In addition, these shoulders were more predominant in MNPs@Oregano and MNPs@Star Anise samples, possibly indicating that surface oxidation was also promoted by *I. verum* and *L. graveolens* extracts, meaning that clove aqueous extract provided better protection against oxidation. Furthermore, the spectra show the vibrational modes of the biomolecules bonded to the surface of the Fe_3_O_4_NPs@polyphenols. Broadband absorption centred at 3400 cm^−1^ is the result of the stretching vibration of the hydroxyl group –OH^60–62^ possibly due to hydrogen bonding between polyphenols.^63^ The peak detected at 1630 cm^−1^ can be attributed to the aromatic ring (C=C) stretching mode and carbonyl groups (C=O).^64–66^ The set of lower frequency bands in the range of 1485–1265 cm^−1^ is consistent with the bending vibrations of methyl (CH_3_) and methylene (CH_2_) groups that are present in eugenol, anethole, and thymol.^65,67^ Moreover, the spectra of MNPs@Star Anise and MNPs@Oregano in the 2980–2820 cm^−1^ signal are related to these functional groups but correspond to the stretching vibrational modes.^62^ The group of bands in the 1170–970 cm^−1^ signal was attributed to the CO bond, and the authors associated this peak with the presence of the methoxy group, alcohols, and ethers.^60,61,66^ Dhar *et al.* (2021) obtained iron oxide nanoparticles coated with *Lathyrus sativus* peel extract with a mean particle size of 17.72 nm.^63^ Dheyab *et al.* (2020) reported a green synthesis route to produce magnetite nanoparticles of *D* ≈ 19 nm using citric acid.^68^ Both studies reported the frequency bands of the Fe–O bond at 586 and 578 cm^−1^ and the hydroxyl group –OH at 3434 and 3384 cm^−1^, respectively. MNPs@Clove, MNPs@Star Anise, and MNPs@Oregano agreed with the Fe–O signal characteristics of magnetite and the –OH bond linked at the surface of the nanoparticles. The presence of hydroxyl groups (–OH) is important because of their ability to reduce metal ions by donating electrons to the metal cations Fe^2+^/Fe^3+^ to promote the formation of Fe_3_O_4_ nanoparticles.^69,70^ Polyphenols play an important role in the mechanism of nanoparticle formation as reducing and stabilizing agents. More specifically, hydroxyl (–OH), carbonyl (C=O), amino (NH_2_), and methoxide (CH_3_O–) groups are the main compounds in plant extracts involved in the reduction of metal ions and stabilization stages of nanoparticle synthesis.^18^

**Fig. 3:**
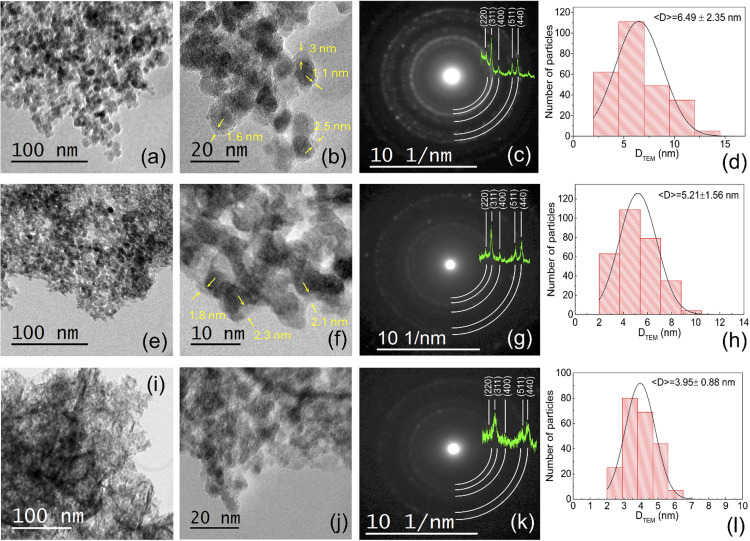
TEM micrographs, SAED patterns, and particle size distributions fitted to a Gaussian function of MNPs@Clove (a–d), MNPs@Star Anise (e–h) and MNPs@Oregano (i–l), respectively. The quasi-spherical morphology of the Fe_3_O_4_NPs@polyphenols and the amorphous surface layer are evident from the TEM micrographs.

The TEM images show the common quasi-spherical morphology of the Fe_3_O_4_NPs@polyphenols, Fig. 3a, e and i. In addition, an amorphous surface layer of thickness *t* ≈ 1–3 nm was observed, capping the MNPs cores, related to the polyphenols present in the aqueous plant extract, as shown in Fig. 3b and f. Particularly, MNPs@Oregano showed a different configuration, with nano-branches uniformly spread among the nanoparticles. The selected area electron diffraction (SAED) analysis exhibited characteristic rings of the diffracting planes (220), (311), (400), (422), (511), and (440) in all samples, in agreement with their respective XRD diffractograms, as shown in Fig. 3c, g and k. The particle size distribution histogram was fitted to a Gaussian function, obtaining mean particle sizes of 6.29 ± 2.35 nm, 5.21 ± 1.56 nm, and 3.95 ± 0.88 nm for MNPs@Clove, MNPs@Star Anise, and MNPs@Oregano, respectively. These crystallite sizes are consistent with the average size estimated from the XRD diffraction peaks. Additionally, the coated nanoparticles exhibited different particle size distribution widths, as MNPs@Clove and MNPs@Star Anise presented wider distributions. This could be ascribed to the structure and length of the polyphenol chains present in aqueous extracts.^2^

**Fig. 4:**
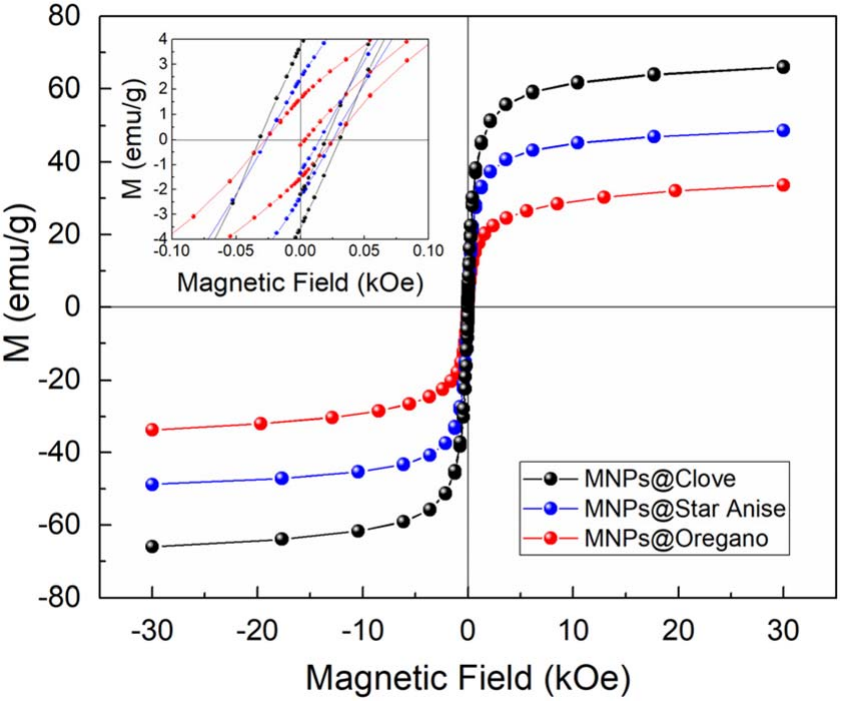
Hysteresis loops of the MNPs@Clove, MNPs@Star Anise, and MNPs@Oregano samples measured at 300 K and a magnetic field of 3 T. Inset graph shows the coercive fields for each synthesis.

The magnetic response *M* (emu g^−1^) as a function of an external magnetic field *H* (kOe) at room temperature (300 K) of all samples exhibited near superparamagnetic performance (Fig. 4), considering that the hysteresis loops demonstrated negligible coercivity and remanence, as expected for nanoparticles of these sizes. The inset graph showed an amplification of −0.1 emu g^−1^ ≤ *H* ≤ 0.1 emu g^−1^ and −4 kOe ≤ *M* ≤ 4 kOe region, where a small contribution of the coercive field *H*_C_, which could originate from the contribution of agglomerated nanoparticles. The values of *H*_C_ were 0.031 kOe, 0.025 kOe, and 0.026 kOe for MNPs@Clove, MNPs@Star Anise and MNPs@Oregano, respectively. The magnetization saturation *M*_S_ values from the de hysteresis loops were 66.07 emu g^−1^ for MNPs@Clove, 48.76 emu g^−1^ for MNPs@Star Anise, and 33.61 emu g^−1^ for MNPs@Oregano. Saturation magnetization, *M*_S_, decreases with size as a result of the increased surface-to-volume ratio of nanoparticles.^71^ Surface effects become more prominent when the size of the nanoparticles decreases due to an increased volume fraction of surface atoms within the entire particle. Consequently, these effects could have an impact on the *M*_S_ value of magnetic nanoparticles, approaching the bulk value when the size increases. Additionally, the organic coating can affect *M*_S_ by acting as an antiferromagnetic barrier, which induces disorder on the surface of the superparamagnetic nanoparticles.^3^

**Fig. 5:**
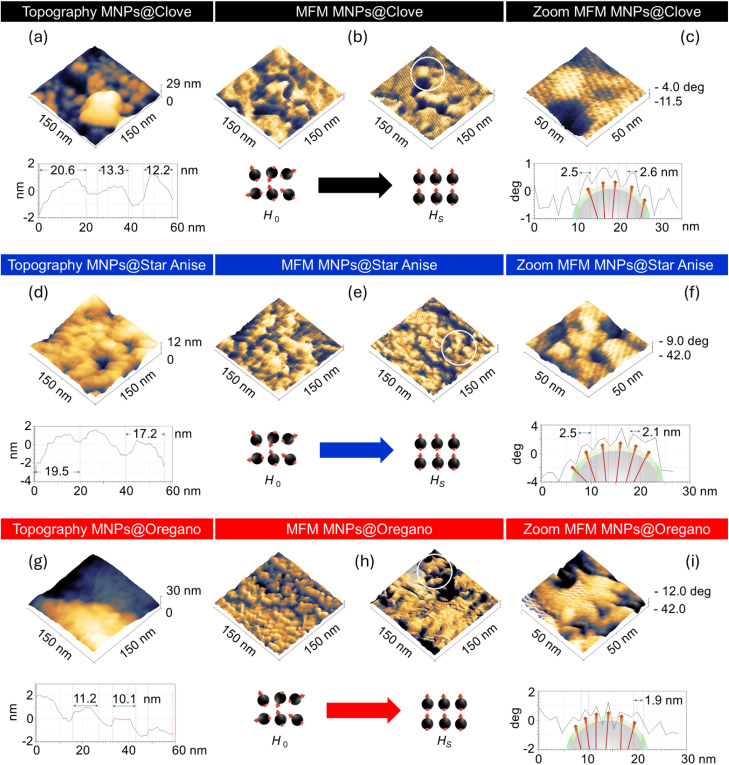
AFM 3D topography of (a) MNPs@Clove, (d) MNPs@Star Anise and (g) MNPs@Oregano with their respective profile sizes. MFM 3D images of magnetic domain responses at initial and under saturation conditions for (b) MNPs@Clove, (e) MNPs@Star Anise and (h) MNPs@Oregano and the zoomed images at 50 × 50 nm in the lift mode (c, f, and i) and their respective profiles.

The surface topography and particle distribution of Fe_3_O_4_NPs@polyphenols were observed using scanning probe microscopy (SPM). The nanoparticle samples were scanned and resolved at areas defined as 150 × 150 nm in tapping mode for topography. The measurements for the MNPs@Clove, MNPs@Star Anise, and MNPs@Oregano samples are shown in Fig. 5a, d, and g, respectively. The 3D images exhibit nanoparticles compressed in a continuum medium. The MNPs@Oregano showed more agglomeration because of its different configuration with a smaller mean particle size and neighboring interaction forces. The topography profiles indicated sizes estimated in the range of 12–20 nm for MNPs@Clove, 17–19 nm for MNPs@Star Anise and 10–11 nm for MNPs@Oregano, considering the overlapping and agglomeration of NPs.

The MFM pictures were tuned in lift mode according to the interaction from the tip with a radius of <40 nm as a large magnet with respect to the surface of small single magnetic domains (SMDs) for NPs in the range of 3.95–6.29 nm verified previously by TEM. The MFM analysis was performed from the initial conditions at *H*_0_ (0) to the applied field saturation *H*_S_ (↑) over the surface of the samples. The magnetic field lines were observed in the MFM contrast at *H*_S_ (↑), as shown in Fig. 5b, e and h. The parallel line suggests an effective uniaxial magnetic response. Fig. 5c, f and i demonstrate the zoom images at 50 × 50 nm in lift mode and their respective SMDs profiles. The 3D images showed the collinear spreading of the magnetic field lines and the directional SMDs with respect to the magnetic dipole moment response. SMDs profiles indicated the magnetic field lines 1.9–2.6 nm condition at *H*_S_ (↑) and (*Z* + Δ*Z*) lift height.^72,73^ The alignment of SMDs is the result of the small magnetic anisotropic energy at superparamagnetic sizes.^74^ The anisotropic energy is given by *E*_a_ = *KV* sin 2*θ*, where *K* is the magnetocrystalline anisotropic constant of the material, *V* is the volume of the nanoparticle, and *θ* is the angle between the magnetization vector and the easy axis of the material. The energy barrier *KV* separates two energetically equivalent axes of anisotropy and prevents a change in the magnetization direction from the spin-up state to the spin-down state.^75^ Nevertheless, this condition only applies to materials with multidomain structures. The theory developed by Kittel showed that the anisotropy energy and domain structure change for ferromagnetic materials with small dimensions and that the optimal configuration corresponds to a single domain magnetized to saturation in one direction. According to Kittel, the critical sizes for the transition from a multidomain to a single-domain configuration are estimated to be approximately 3 × 10^−7^ m for thin films and 2 × 10^−8^ m for nanoparticles.^76^ At these critical sizes, the relative contributions of the different energy components to the total energy are modified, and surface-related energies become more dominant than volume energies; consequently, it is more favourable to energetically support the external magnetostatic energy of the single-domain state than to create a domain wall.^77^ The lowest-energy state of a single-domain configuration depends on the shape and dimensions of the specimen, exhibiting distinct behavior in thin films and small particles.^72,76^ Furthermore, the nearly closed hysteresis loops observed at room temperature, defined by low coercive fields (25–31 Oe), suggest that thermal activation significantly reduces the effective anisotropy barrier *KV*. Therefore, although the individual nanoparticles are single-domain structures, the low-coercive fields indicate the anisotropic energy barrier, and the magnetic moments of the superparamagnetic nanoparticles can freely move away from the easy axis owing to the thermal energy, *K*_B_*T*, which exceeds the anisotropy energy, according to the Néel theory.^78^ Therefore, at room temperature, the anisotropic magnetic contrast observed in MFM under an applied field is more accurately attributed to a combination of intrinsic magnetocrystalline anisotropy and collective effects^79^ related to dipolar interactions and assembly-induced shape anisotropy, especially in the branched and quasi-2D structures present in the oregano-coated sample. MNPs@Oregano nanoparticles assembled into nano-branch structures oriented in the (*x*–*y*) plane as 2D assemblies, as observed in the TEM micrographs. This change in configuration could cause collective magnetic interactions similar to those observed in thin-film systems due to dipole interactions.^80^ The MFM zoom exhibited the uniaxial response of the SMDs in lift mode, as shown in Fig. 5c, f and i. The values in the shift phase were −4.0 to −11.5 deg for MNPs@Clove and −9.0 to −42 deg for MNPs@Star Anise, and −12 to −42 deg for MNPs@Oregano. MFM images showed a relative phase contrast that originated from variations in the phase shift due to the interaction between the magnetized tip and the stray magnetic field generated by the nanoparticles. The relative phase contrast differs between samples. MNPs@Clove exhibited a smaller phase contrast, which can be attributed to weaker variations in the local magnetic interaction. Moreover, MNPs@Star Anise and MNPs@Oregano showed greater and comparable phase contrasts, indicating stronger variations in the local magnetic response of nanoparticle aggregates. The differences in the phase contrast of the three samples suggest that the arrangement of nanoparticles and the organic coating could affect the local magnetic interactions. Considering the nanoparticle size range below 10 nm, it is important to note that the individual magnetic response of nanoparticles could not be determined easily by MFM due to the limited magnetic signal associated with these small magnetic volumes. In the case of NPs in powder, the phase contrast observed in the MFM images is more likely to be related to the collective magnetic response of nanoparticle aggregates than to the magnetic domains of isolated particles. The profiles indicated the normal orientation of the magnetic domains at 90° and flux lines from SMDs obtained: 2.5–2.6 nm for MNPs@Clove, 2.1–2.5 nm for MPNs@Star Anise, and 1.9 nm for MNPs@Oregano (Fig. 5c, f and i, respectively). Therefore, the Fe_3_O_4_NPs@polyphenols exhibited an effective uniaxial response under an applied magnetic field, which is an important feature for directional nanocores and their possible future applications as nanocarriers.^81^

## Conclusions

In this study, we proposed an eco-friendly synthesis route for producing magnetite nanoparticles *via* a direct polyphenol coating method using the aqueous extracts of clove (*Syzygium aromaticum*), star anise (*Illicium verum*), or Mexican oregano (*Lippia graveolens*). Polyphenols present in aqueous extracts promoted the reduction of metal ions and acted as stabilizing agents. The modified co-precipitation method offers facile functionalization of Fe_3_O_4_ with superparamagnetic sizes coated with organic layers in the range of *t* ≈ 1–3 nm. The inverse spinel structure of magnetite was confirmed in the Fe_3_O_4_NPs@polyphenols as well, with a small lattice distortion related to surface oxidation, suggesting the presence of γ-Fe_2_O_3_. The organic bonds associated with functional groups –OH, CH_3_, CH_2_, CO, and aromatic compounds were identified by FTIR analysis, and the organic layer of thickness *t* ≈ 1–3 nm was observed *via* TEM, confirming the affinity of the phenol groups to be attached to Fe ions on the surface of the synthesized nanoparticles. The magnetization hysteresis loops indicated that all synthetized nanoparticles are in the superparamagnetic regime and have single-domain behavior. In addition, the uniaxial magnetic response of the SMDs was verified under magnetization saturation *H*_S_(↑), indicating directional performance. The results showed that Fe_3_O_4_NPs@polyphenols are potential candidates for biomedical encapsulation and applications, as their surface hydroxyl groups (–OH) provide active binding sites for the conjugation of drugs, enzymes, antibodies, and other biomolecules. These properties make functional SPIONs promising alternatives for several future biological studies owing to their development in therapeutic strategies, such as magnetic hyperthermia and nanocarriers for drug delivery. Nonetheless, further biological studies, including cytotoxicity, colloidal stability, and the evaluation of physiological media, would require evaluating their potential in such applications.

## Author contributions

Conceptualization: N. E. M.-C and J. S.-S; methodology and experimental work: N. E. M.-C and J. S.-S; formal analysis and investigation: N. E. M.-C and J. S.-S; writing – original draft preparation: N. E. M.-C and J. S.-S; writing – review and editing: N. E. M.-C, M. D. C.-T. and J. S.-S.; funding acquisition: J. S.-S.; resources: M. D. C.-T and J. S.-S.; and supervision: J. S.-S. All authors have read and approved the final manuscript.

## Conflicts of interest

There are no conflicts to declare.

## Data Availability

All data generated or analyzed during this study are included in the manuscript.
